# Prostacyclin and thromboxane in benign and malignant breast tumours.

**DOI:** 10.1038/bjc.1986.194

**Published:** 1986-09

**Authors:** G. M. Laekeman, I. B. Vergote, G. M. Keersmaekers, J. Heiremans, C. F. Haensch, G. de Roy, F. L. Uyttenbroeck, A. G. Herman

## Abstract

6-keto-PGF1 alpha and thromboxane B2 were determined by radioimmunoassay in 37 extracts of breast carcinomata, 8 fibroadenomata, 12 sclerocystic-disease specimens and 51 normal breast tissues. More prostanoids were extracted from carcinomata than from normal specimens, fibroadenomata or sclerocystic-disease tissues (P less than 0.05). The 6-keto-PGF1 alpha/TXB2 ratio was higher in carcinomata than in normal tissues and fibroadenomata (P less than 0.05) but was not significantly different from the ratio in sclerocystic disease. The prostaglandin levels and the 6-keto-PGF1 alpha/TXB2 ratios from carcinomata did not correlate significantly with age, tumour size, differentiation, lymph node status, nuclear-cytoplasmic ratio, host cell reaction, mast cells, necrosis, elastosis, fibrosis or blood vessel density. Lower nuclear density was associated with lower 6-keto-PGF1 alpha/TXB2 ratios (P = 0.01) whereas the latter value was higher when infiltration was lower (P = 0.03). There was a positive correlation between mitotic index and the 6-keto-PGF1 alpha/TXB2 ratio (P = 0.04). Cumulation of variables revealed lower prostanoid ratios in tumours greater than 2 cm without lymph node metastasis then tumours less than 2 cm with lymph node metastasis (P = 0.04). A first follow-up (14 months) showed a higher 6-keto-PGF1 alpha/TXB2 ratio in patients who developed metastasis (P = 0.04). Our study does not confirm the hypothesis that high prostacyclin levels are a good prognostic index in breast cancer.


*Br. J. Cancer* (1986) **54**, 431–437

### Prostacyclin and thromboxane in benign and malignant breast tumours

G.M. Laekeman[1], I.B. Vergote[2], G.M. Keersmaekers[2], J. Heiremans[1], C.F. Haensch[2], G. de Roy[2], F.L. Uyttenbroeck[2] & A.G. Herman[1]

[1]*Division of Pharmacology and* [2]*Department of Gynecology and Obstetrics, and Pathology, St. Camillus Hospital, University of Antwerp, Universiteitsplein, 1, B2610 Wilrijk, Belgium.*

**Summary** 6-keto-$PGF_{1\alpha}$ and thromboxane $B_2$ were determined by radioimmunoassay in 37 extracts of breast carcinomata, 8 fibroadenomata, 12 sclerocystic-disease specimens and 51 normal breast tissues. More prostanoids were extracted from carcinomata than from normal specimens, fibroadenomata or sclerocystic-disease tissues ($P<0.05$). The 6-keto-$PGF_{1\alpha}/TXB_2$ ratio was higher in carcinomata than in normal tissues and fibroadenomata ($P<0.05$) but was not significantly different from the ratio in sclerocystic disease. The prostaglandin levels and the 6-keto-$PGF_{1\alpha}/TXB_2$ ratios from carcinomata did not correlate significantly with age, tumour size, differentiation, lymph node status, nuclear-cytoplasmic ratio, host cell reaction, mast cells, necrosis, elastosis, fibrosis or blood vessel density. Lower nuclear density was associated with lower 6-keto-$PGF_{1\alpha}/TXB_2$ ratios ($P=0.01$) whereas the latter value was higher when infiltration was lower ($P=0.03$). There was a positive correlation between mitotic index and the 6-keto-$PGF_{1\alpha}/TXB_2$ ratio ($P=0.04$). Cumulation of variables revealed lower prostanoid ratios in tumours >2 cm without lymph node metastasis then tumours <2 cm with lymph node metastasis ($P=0.05$). A first follow-up (14 months) showed a higher 6-keto-$PGF_{1\alpha}/TXB_2$ ratio in patients who developed metastasis ($P=0.04$). Our study does not confirm the hypothesis that high prostacyclin levels are a good prognostic index in breast cancer.

Honn *et al.* (1981) found that prostacyclin ($PGI_2$) had a beneficial influence against metastasis of B16 amelanotic melanoma tumours in mice, whereas inhibitors of $PGI_2$ synthesis enhanced the number of metastases. Other authors tested the antimetastatic potency of acetylsalicylic acid, indomethacin, dipyridamole, flurbiprofen, benorylate, heparin and warfarin (Gasic *et al.*, 1973; Elias *et al.*, 1973; Lione & Bosman, 1978; Bennett, 1982). Most of these studies are done on animals and showed no clearcut results.

Thromboxane (TX) $A_2$ is often a physiological antagonist of $PGI_2$ and an imbalance between them can disturb the (anti)haemostatic system. The anti-aggregating properties of nonsteroidal anti-inflammatory drugs (NSAID) can be explained by a stronger inhibition of the platelet cyclo-oxygenase in comparison with that of the vessel wall. As a result the release of TX will be lowered and aggregation will be blocked (Bunting *et al.*, 1983). Sloane *et al.* (1981) showed that some tumours are able to release cathepsin B. This enzyme stimulates the synthesis of TX and is produced in a variant of B16 melanoma which has high metastatic activity.

In order to study a possible prognostic value of the $PGI_2$/TX ratio in breast cancer, the stable hydrolysis products of $PGI_2$ (6-keto-$PGF_{1\alpha}$) and $TXA_2$ ($TXB_2$) were determined by RIA. 6-keto-$PGF_{1\alpha}$ and $TXB_2$ levels were examined in relation to the size of the tumour, axillary lymph node status, lymphatic vessel permeation, differentiation of the tumour, mitotic index density of nuclei of tumour cells, and age of the patient.

PG production can be influenced by inflammatory processes (Humes *et al.*, 1977; Brune *et al.*, 1978), and therefore the number of host-derived cells and the amount of necrosis was evaluated. Also the density of blood vessels was estimated as they could be a major source of 6-keto-$PGF_{1\alpha}$ (Moncada *et al.*, 1976), and platelets contribute considerably to the amounts of $TXB_2$ measured (Hamberg *et al.*, 1975).

### Materials and methods

We obtained 108 specimens from 67 patients who underwent surgery for a breast lump. Each specimen was divided into two representative parts and prepared as described earlier by Vergote *et al.* (1985). The tissues were immediately immersed either in acetone cooled by solid $CO_2$ (−70°C) for 6-keto-$PGF_{1\alpha}$ or $TXB_2$ analysis, or in Bouin's liquid for histopathological examination. The tissue samples for prostanoid investigation were stored at −30°C until radioimmunoassay was performed.

Correspondence: G.M. Laekeman
Received 8 November 1985; and in revised form 23 April 1986.

© The Macmillan Press Ltd., 1986

Thirty-five tumours were diagnosed as primary breast cancer (patient age range: 36–82; mean 64). The patients were classified according to the pathological TNM system (UICC, Livre de Poche), pTlaNo 9; pTlaNla 1; pTlaNlb 4; pTlaNx 2; pT2aNo 4; pT2aNla 2; pT2aNlb 4; pT2aNx 2; pT3aNo 2; pT4bNx 1. No patient had overt metastases at the time of surgery and none was under current treatment with non-steroidal inflammatory drugs or corticosteroids. Five local relapses (ductal carcinomata) and one cellular intracanicular fibroadenoma were included. Twenty tumours were benign: 8 fibroadenomata (age range: 17–47, mean: 34) and 12 sclerocystic-disease specimens (age range: 37–70, mean: 49). We investigated also histologically confirmed normal breast tissue of patients with malignant tumours and fibroadenomata. Furthermore, 12 specimens of patients who had neither benign nor malignant tumours were studied. The age of the patients and the tumour size at anatomopathological examination were recorded. The amounts of 6-keto-$PGF_{1\alpha}$ and $TXB_2$ were expressed as $ng\,mg^{-1}$ protein and from these values the 6-keto-$PGF_{1\alpha}/TXB_2$ ratio was calculated.

### Histopathology

The slides were independently reviewed by two of the authors and re-evaluated by a senior pathologist. In case of discordance, the results were not included in this study.

Tumours were classified according to the methodology used earlier (Vergote *et al.*, 1985). Subdivision of histopathological variables is shown in Table III.

### *Radioimmunoassay (RIA)*

For the extraction of prostaglandins and the protein measurement the procedure of Vergote *et al.* (1985) was used. Acetone was evaporated under nitrogen and the weight of the tissue determined. Tris buffer (50 mM, pH = 8.0 at 25°C) was added ($3\,ml\,g^{-1}$ tissue) and sonicated for 90 min (Bransonic). Ice was regularly added to the bath fluid. The supernatant was separated from the tissue after centrifugation at 10,000 *g* (Eppendorf centrifuge). RIA was performed directly on the supernatant according to Granström and Kindahl (1978). The antisera were raised in rabbits. Cross reactivities on the 50% binding level of the curve were: for the 6-keto-$PGF_{1\alpha}$-antiserum: $PGF_{1\alpha}$, 1%; 15-HETE (hydroxyeicosatetraenoic acid), 0.01%; 15-HPETE (hydroperoxyeicosatetraenoic acid), 0.01%; $PGE_2$, 15-keto-$PGE_2$, $TXB_2$ and AA (arachidonic acid) <0.01%; for the $TXB_2$ antiserum: $PGD_2$, 8.9%; $PGF_{2\alpha}$, 1%; $PGE_2$, 0.9%; 6-keto-$PGF_{1\alpha}$, 0.1%; 15-keto-13,14-dihydro-$PGF_{2\alpha}$, AA, 15-HETE and 15-HPETE, <0.01%.

The extraction recoveries for 6-keto-$PGF_{1\alpha}$ were $112 \pm 10\%$ (mean ±SE: n = 3) and for $TXB_2$ $97 \pm 16$ (mean ± SE; n = 3). The intra-assay variation coefficient for RIA of 6-keto-$PGF_{1\alpha}$ and $TXB_2$ were $15 \pm 1\%$ (n = 103) and $13 \pm 1\%$ (n = 111) respectively.

### *Reagents*

$TXB_2$ and 6-keto-$PGF_{1\alpha}$ (Upjohn), ($^3H$)-radiolabelled 6-keto-$PGF_{1\alpha}$ and ($^3H$)-$TXB_2$ (NEN). Tris buffer was made with trizma base (Sigma) and HCl (Merk p.a.).

### *Statistical analysis*

Nonparametric statistical canalysis was used to compare two (Wilcoxon test) or more groups (Kruskal & Wallis test; Sokal & Rohlf, 1981). Correlation coefficients were calculated by linear regression.

### Results

### *PG levels in relation to the histopathological groups*

6-keto-$PGF_{1\alpha}$ levels were higher in carcinomata (CA) than in normal breast tissue (N), fibroadenomata (FA) and sclerocystic disease (SCD) ($P = 0.0003$, Kruskal & Wallis). In FA, N and SCD the levels did not differ significantly ($P = 0.17$, Kruskal & Wallis). CA-$TXB_2$ levels were significantly higher in comparison with the other groups ($P = 0.05$, Kruskal & Wallis). The differences between N, FA and SCD were not significant ($P = 0.83$, Kruskal & Wallis). The 6-keto-$PGF_{1\alpha}/TXB_2$ ratio in CA was higher than in N and FA ($P = 0.002$, Kruskal & Wallis) which were similar ($P = 0.39$). SCD and CA also gave similar ratios ($P = 0.67$). These results are summarized in Table I and Figure 1. When local relapses (n = 5) were calculated separately, 6-keto-$PGF_{1\alpha}$, $TXB_2$ and the 6-keto-$PGF_{1\alpha}/TXB_2$ ratio were respectively, median (limit values): 12.7 (0.6–15.0) $ng\,mg^{-1}$ protein, 2.5 (1.8–3.4) $ng\,mg^{-1}$ protein and 4.0 (0.3–7.3). They were similar to the ductal carcinomata ($P = 0.95$; $P = 0.40$ and $P = 0.28$ respectively).

### *Histological type and differentiation*

The infiltrating ductal carcinomata composed the substantial group. Statistical comparison between all the groups was difficult because some contained very few cases (Table II). We divided the tumours into two groups: undifferentiated and some degree of differentiation (small, noderate or high). Both

**Table I** PG-levels in relation to the pathology

| *Pathology* | *n* | *ng 6-keto-PGF$_{1\alpha}$ mg$^{-1}$ protein median (semiquartiles)* | *ng TXB$_2$ mg$^{-1}$ protein median (semiquartiles)* | *6-keto-PGF$_{1\alpha}$/TXB$_2$ median (semiquartiles)* |
|---|---|---|---|---|
| CA | 37 | 4.4 (1.7–14.3) | 0.6 (0.4–2.8) | 4.6 (3.2–9.2) |
| N | 51 | 1.2 (0.4–3.3) | 0.4 (0.2–0.8) | 2.6 (1.4–5.2) |
| FA | 8 | 0.3 (0.2–1.8) | 0.4 (0.2–0.5) | 1.8 (0.7–4.9) |
| SCD | 12 | 1.7 (0.9–3.5) | 0.4 (0.2–1.0) | 4.3 (2.7–8.7) |

CA = carcinomata; N = normals; FA = fibroadenomata; SCD = sclerocystic disease.

**Figure 1** Individual 6-keto-PGF$_{1\alpha}$/TXB$_2$ values in the pathological groups: CA (n = 37; carcinomata), N (n = 51; normal breast tissue), FA (n = 8; fibroadenomata), SCD (n = 12; sclerocystic disease). Medians and semiquartiles are indicated.

**Table II** PG-levels in relation to the histological type

| *Type* | *n* | *ng 6-keto-PGF$_{1\alpha}$ mg$^{-1}$ protein* | *ng TXB$_2$ mg$^{-1}$ protein* | *6-keto-PGF$_{1\alpha}$/TXB$_2$* |
|---|---|---|---|---|
| Infiltrating ductal carcinoma | 23 | 4.4 (2.3–16.6)[a] | 1.0 (0.3–3.4)[a] | 4.6 (3.3–9.6)[a] |
| Lobular | 2 | 5.1–8.1 | 0.4–0.5 | 12.8–15.2 |
| Comedo | 4 | 0.4–2.2[b] | 0.1–0.7[b] | 0.6–8.33[b] |
| Medullary | 1 | 1.4 | 0.4 | 4.0 |
| Mucoid | 1 | 7.7 | 3.6 | 2.1 |

[a]Median (semiquartiles); [b]Limit values groups showed similar 6-keto-$PGF_{1\alpha}$, $TXB_2$ and 6-keto-$PGF_{1\alpha}/TXB_2$ levels (Table III).

*Lymph node metastasis and lymphatic vessel permeation*

The median amount of 6-keto-$PGF_{1\alpha}$ was slightly higher in tumour extracts from patients without lymph node metastasis ($P=0.23$). Median $TXB_2$ amounts and the 6-keto-$PGF_{1\alpha}/TXB_2$ ratio were comparable.

The groups without lymphatic vessel permeation had more 6-keto-$PGF_{1\alpha}$ ($P=0.04$), but the $TXB_2$ values and the 6-keto-$PGF_{1\alpha}/TXB_2$ ratio were similar (Table III).

*Size and density of nuclei of carcinoma cells*

Tumours with moderate density had significantly less 6-keto-$PGF_{1\alpha}$ ($P=0.04$) and $TXB_2$ ($P=0.01$) than those with low or high density. Higher 6-keto-$PGF_{1\alpha}/TXB_2$ ratios were found in moderate and high density groups ($P=0.01$; Table III).

Carcinomata with large nuclei tended to yield less $TXB_2$ than those having moderate size nuclei ($P=0.18$; Table III).

*Mitotic index*

There was at most a weak correlation between the number of mitoses per high power field (HPF) and the 6-keto-$PGF_{1\alpha}$ or $TXB_2$ levels ($r=-0.167$, $P=0.18$ and $-0.212$, $P=0.13$ respectively), but a positive correlation occurred with the 6-keto-$PGF_{1\alpha}/TXB_2$ ratio ($r=0.33$, $P=0.04$).

*Nuclear and cellular polymorphism and the nuclear cytoplasmic ratio*

Tumours with a low nuclear and cellular polymorphism showed at most a weak tendency to higher 6-keto-$PGF_{1\alpha}$ levels ($P=0.19$) no other relationships were seen.

*Host cell reaction, necrosis and mast cells*

Host cell reaction, necrosis and presence of mast cells did not correlate with the amounts of extracted prostanoids.

*Elastosis, fibrosis and infiltration*

No significant tendencies were observed between PG-levels and elastosis or fibrosis. Infiltration was inversely related to the 6-keto-$PGF_{1\alpha}/TXB_2$ ratio ($P=0.03$), but no significant differences were seen for the 6-keto-$PGF_{1\alpha}$ or $TXB_2$ (Table III).

*Blood vessel density*

Presence of blood vessels did not correlate with prostanoid yields in the tumour biopsies. the 6-keto-$PGF_{1\alpha}/TXB_2$ ratio even tended to be lower when more blood vessels were present ($P=0.18$; Table III).

*Age*

No correlations between prostanoids and age were seen: 6-keto-$PGF_{1\alpha}$, $r=-0.023$ ($P=0.45$), $TXB_2$, $r=0.157$ ($P=0.20$), 6-keto-$PGF_{1\alpha}/TXB_2$ $r=0.179$ ($P=0.20$).

*Cumulation of variables and follow up*

Tumour size showed little or no relationship to tissue prostanoids: 6-keto-$PGF_{1\alpha}$ $r=-0.182$ ($P=0.16$), $TXB_2$ $r=-0.126$ ($P=0.41$), 6-keto-$PGF_{1\alpha}/TXB_2$ $r=-0.038$ ($P=0.42$). Tumours with strong metastatic potential (<2 cm and lymph node metastasis, n=5) had a median 6-keto-$PGF_{1\alpha}/TXB_2$ ratio of 3.8 (2.0–9.4), which was higher ($P<0.05$) than with tumours having a relatively weak metastatic potential (>2 cm and no lymph node metastasis, n=7; 2.8 (0.5–4,6)).

A preliminary analysis of 19 patients with a follow-up of 14 months revealed metastases in 5 patients. These 5 patients had a median 6-keto-$PGF_{1\alpha}/TXB_2$ ratio of 9.2 (4.0–15.3) which was higher than the ratio of nonmetastatic patients 4.8 (1.6–13.5) ($P=0.04$).

### Discussion

Little is known about the role of $PGI_2$ and TX in human malignant tumours, and only 2 studies on extracted breast tissues have been published.

Karmali *et al.* (1983) measured 6-keto-$PGF_{1\alpha}$ and $TXB_2$ in 24 breast tumours and expressed their results as log ng $g^{-1}$ wet weight. They obtained more $TXB_2$ from large tumours and those with lymph node metastasis. These results were interpreted as supporting the findings of Honn (1981) that a higher $TXA_2/PGI_2$ ratio has a worse prognosis in terms of metastasis.

However, Karmali *et al.* (1983) did not give any data about the 6-keto-$PGF_{1\alpha}/TXB_2$ ratio which is important in the regulation of blood platelet aggregation. Furthermore, the size of the tumour is not necessarily an indication of the metastatic potential. In addition, Karmali *et al.* (1983) observed no correlation between $TXB_2$ levels and metastasis. Aitokallio-Tallberg *et al.* (1985) studied the *in vitro* production of 6-keto-$PGF_{1\alpha}$ and $TXB_2$ by 23 breast tumour tissues, but as their investigations were directed towards steroid receptor status

**Table III** Prostanoid levels and anatomopathological variables

| *Variable (n)* | *ng 6-keto-$PGF_{1\alpha}$ $mg^{-1}$ prot. median (limit values)* | *P* | *ng $TXB_2$ $mg^{-1}$ prot. median (limit values)* | *P* | *6-keto-$PGF_{1\alpha}$ $TXB_2$ median (limit values)* | *P* |
|---|---|---|---|---|---|---|
| Differentiation | | | | | | |
| no diff. (15) | 5.0 (0.6–27.4) | 0.44 | 0.5 (0.1–17.0) | 0.71 | 4.3 (0.3–17.5) | 0.68 |
| diff. (16) | 4.4 (0.9–62.9) | | 0.8 (0.1–12.8) | | 5.0 (2.4–14.4) | |
| Lymph node metastasis | | | | | | |
| positive (11) | 3.9 (0.7–45.3) | 0.23 | 0.5 (0.3–6.5) | 0.70 | 4.8 (2.8–10.0) | 0.89 |
| negative (15) | 5.4 (0.5–62.9) | | 0.7 (0.1–12.8) | | 4.3 (1.8–9.2) | |
| Lymphatic vessel permeation | | | | | | |
| positive (23) | 4.0 (0.6–62.9) | 0.04[a] | 0.5 (0.1–17.0) | 0.17 | 4.7 (0.3–17.5) | 0.53 |
| negative (8) | 9.3 (4.3–29.9) | | 2.1 (0.3–9.6) | | 5.2 (3.1–15.2) | |
| Density of nuclei of carcinoma cells | | | | | | |
| low (8) | 12.7 (1.4–62.9) | 0.04[a] | 3.5 (0.4–12.8) | 0.01[a] | 2.9 (2.1–4.6) | 0.01[a] |
| moderate (16) | 4.0 (0.6–16.7) | | 0.4 (0.1–2.7) | | 6.1 (0.3–17.5) | |
| high (7) | 7.7 (2.2–27.4) | | 0.8 (0.3–17.0) | | 7.0 (1.6–14.4) | |
| Size of nuclei of carcinoma cells | | | | | | |
| small (3) | 8.1 (0.6–14.3) | 0.44 | 0.5 (0.2–1.8) | 0.18 | 7.7 (0.3–15.2) | 0.34 |
| moderate (23) | 5.0 (0.9–27.4) | | 0.8 (0.1–17.0) | | 4.4 (1.6–17.5) | |
| large (5) | 4.0 (1.2–8.3) | | 0.4 (0.3–0.9) | | 9.7 (4.6–13.4) | |
| Nuclear and cellular polymorphism | | | | | | |
| low (8) | 11.2 (0.6–62.9) | 0.19 | 1.2 (0.2–17.0) | 0.44 | 5.1 (0.3–15.2) | 0.98 |
| moderate (15) | 4.4 (0.9–29.9) | | 0.8 (0.1–11.2) | | 4.4 (2.4–17.5) | |
| high (8) | 3.1 (1.2–8.3) | | 0.4 (0.3–3.6) | | 4.9 (2.1–13.4) | |
| Nuclear cytoplasmic ratio | | | | | | |
| low (4) | 5.0 (1.2–16.6) | 0.93 | 1.6 (0.3–3.6) | 0.74 | 4.9 (2.1–6.1) | 0.77 |
| moderate (20) | 4.3 (0.9–62.9) | | 0.7 (0.1–17.0) | | 4.2 (1.6–17.5) | |
| high (6) | 6.6 (0.6–24.6) | | 0.4 (0.2–5.3) | | 5.1 (0.3–15.2) | |
| Host cell reaction | | | | | | |
| low (19) | 5.0 (0.6–62.9) | 0.59 | 0.8 (0.1–12.8) | 0.39 | 4.0 (0.3–17.5) | 0.67 |
| moderate (11) | 4.2 (1.0–27.4) | | 0.5 (0.2–17.0) | | 5.1 (1.6–13.4) | |
| Mast cells | | | | | | |
| positive (11) | 4.1 (1.2–62.9) | 0.56 | 0.7 (0.3–12.8) | 0.77 | 4.6 (2.1–13.4) | 0.34 |
| negative (20) | 5.4 (0.6–29.9) | | 0.6 (0.1–17.0) | | 5.1 (0.3–17.5) | |
| Necrosis | | | | | | |
| positive (15) | 4.0 (1.2–26.5) | 0.21 | 0.5 (0.1–11.2) | 0.59 | 4.8 (2.1–17.5) | 0.97 |
| negative (16) | 7.9 (0.6–62.9) | | 0.8 (0.1–17.0) | | 4.4 (0.3–15.2) | |
| Elastosis | | | | | | |
| positive (21) | 5.4 (1.4–62.9) | 0.32 | 0.9 (0.2–12.8) | 0.57 | 5.1 (0.3–17.5) | 0.13 |
| negative (10) | 4.3 (0.6–27.4) | | 0.6 (0.1–17.0) | | 3.2 (2.4–13.4) | |
| Fibrosis | | | | | | |
| negative (2) | 10.4–15.0 | 0.52 | 2.9–3.4 | 0.40 | 3.6–4.4 | 0.55 |
| low (6) | 5.9 (0.6–8.3) | | 0.7 (0.3–1.8) | | 9.4 (0.3–15.2) | |
| moderate (16) | 4.4 (1.2–62.9) | | 0.7 (0.1–17.0) | | 4.6 (1.6–17.5) | |
| high (7) | 4.2 (0.9–29.9) | | 0.5 (0.1–9.6) | | 8.1 (2.1–10.1) | |
| Infiltration | | | | | | |
| low (3) | 10.4 (1.7–15.0) | 0.92 | 2.9 (0.4–3.4) | 0.49 | 4.4 (3.6–4.6) | 0.03* |
| moderate (14) | 4.4 (0.9–26.5) | | 0.5 (0.1–11.2) | | 8.0 (2.4–17.5) | |
| high (14) | 5.0 (0.6–62.9) | | 1.0 (0.2–17.0) | | 3.2 (0.3–10.0) | |
| Blood vessel density | | | | | | |
| low (14) | 5.3 (0.9–29.9) | 0.49 | 0.8 (0.1–9.6) | 0.38 | 8.0 (2.1–17.5) | 0.18 |
| moderate (8) | 6.0 (1.2–27.4) | | 0.9 (0.3–17.0) | | 3.7 (1.6–15.2) | |
| high (7) | 2.2 (0.6–62.9) | | 0.4 (0.2–12.8) | | 4.8 (0.3–13.4) | |

[a]Significantly different they did not include control tissue superfusion in their protocol. The prostanoid yields were similar from metastatized and non-metastatized cancers (follow-up of at least 3 years).

We studied the PG-levels in the tumours at the time of resection. Treatment with acetone at −70°C inactivated the tumour enzymes and stopped the conversion of arachidonic acid to prostaglandins (Vergote *et al.*, 1985). Prostanoids were measured in the tumour extracts, but the recoveries were about 100%, we can consider the RIA-results as reflecting the prostanoid production at the time of resection. Since the surgical manipulation might induce $PGI_2$ and $TXA_2$ production, we also analyzed normal breast tissue taken from the neighbourhood of the cancer. The tumour specimen was always taken first, so that artefactual prostanoid might be higher in the normal tissue because of the longer trauma or lower because of prostanoid metabolism. For example 6,15-diketo 13,14-dihydro $PGF_{1\alpha}$ is formed from $PGI_2$ in plasma and vascular tissue (Peskar *et al.*, 1980).

However, continued enzymatic conversion would be blocked by the acetone treatment.

Non-malignant sources of the tumour prostanoids are the host cells, but no correlation was found between the amounts of the prostanoids and the host cell reaction or the presence of mast cells. Furthermore, the 6-keto-$PGF_{1\alpha}$ and $TXB_2$ tissue levels did not correlate with the density of blood vessels.

We preferred to express the prostanoid yields in $ng\,mg^{-1}$ protein, rather than to use $ng\,g^{-1}$ wet or dry weight, in an attempt to reduce the variation of the results. One of the main conclusions from our study is that carcinomata had higher median 6-keto-$PGF_{1\alpha}$ and $TXB_2$ levels than N, FA or SCD, but even so, the ranges overlapped considerably.

As can be seen from Table III, most of the differences were not significant, but a high metastatic potential and mitotic index correlated with higher 6-keto-$PGF_{1\alpha}$/$TXB_2$ ratios. Furthermore, with 42 comparisons, 2 would be expected by chance to show $P$ values $<0.05$ when no difference really exists.

No firm conclusions can yet be made, although follow-up over 14 months revealed a higher 6-keto-$PGF_{1\alpha}$/$TXB_2$ ratio in patients with metastasis. These data should be interpreted very cautiously, as the number of patients evaluated is low and the follow-up period is limited. Nevertheless, the histopathology results do not support the suggestion of Karmali *et al.* (1983), for a protective action of prostacyclin against metastasis in human breast cancer, or the hypothesis of Honn (1981) that $TXA_2$ promotes metastasis.

A common observation by several authors is the greater prostaglandin production by malignant breast tumours compared with normal breast tissues. This conclusion is independent of the prostaglandin measured or the ways of performing the incubations, extractions or measurements (Bennett *et al.*, 1975; Kibbey *et al.*, 1979; Greaves *et al.*, 1980; Rolland *et al.*, 1980; Malachi *et al.*, 1981; Campbell *et al.*, 1983; Karmali *et al.*, 1983). The relationship between high prostaglandin levels and malignancy of breast tumours could lead to the systematic incorporation of cyclo-oxygenase inhibitors (NSAID) in cancer therapy. However, when histopathological prognostic variables of malignant tissues are examined, the prognosis seems to be better when the $PGF_{2\alpha}$ levels were higher (Vergote *et al.*, 1985). Although this was not found for 6-keto-$PGF_{1\alpha}$ and $TXB_2$ in our present study, the mitotic index, prognosis and the first follow-up results indicate that tumour 6-keto-$PGF_{1\alpha}$ at the time of surgery was often higher in patients with bad prognosis or metastasis. Using NSAID could theoretically worsen the prognosis, by depressing the 6-keto-$PGF_{1\alpha}$ levels, but we do not know what effect this would have on the 6-keto-$PGF_{1\alpha}$/$TXB_2$ ratio. Therefore we can neither support nor recommend the use of NSAID in the treatment of breast cancer, particularly since other actions of prostaglandins, e.g. in immunological functions, may also be affected.

The authors wish to thank Dr J. Vanderheyden, Dr G. Albertyn, Dr J. Verkinderen, Dr E. Schatteman, Dr P. Meulyzer, Dr H. Wauters, Dr P. Dalemans and Dr J. Van Wiemeersch of the Department of Gynaecology and Obstetrics, St. Camillus Hospital, University of Antwerp for kindly providing the clinical material; Miss A. Van Hoydonck for technical assistance and Miss L. Van den Eynde for the typewriting.

### References

AITOKALLIO-TALLBERG, A., KARKKAINEN, J., PANTZAR, P., WAHLSTROM, T. & YLIKORKALA, O. (1985). Prostacyclin and thromboxane in breast cancer: Relationship between steroid receptor status and medroxyprogesterone acetate. *Br. J. Cancer*, **51**, 671.

BENNETT, A., McDONALD, A., SIMPSON, J. & STAMFORD, I. (1975). Breast cancer, prostaglandins and bone metastasis. *Lancet*, **i**, 1218.

BENNETT, A. (1982). Effect of prostaglandin synthesis inhibitors on tumour growth *in vivo*. In: *Prostaglandins and Cancer* (Eds. Powles *et al.*) Alan R. Liss Inc., New York. p. 759.

BRUNE, K., KALIN, G. & PESKAR, B. (1978). Pharmacological control of prostaglandin and thromboxane release from macrophages. *Nature*, **274**, 261.

BUNTING, S., MONCADA, S. & VANE, J. (1983). The prostacyclin-thromboxane balance, pathophysiological and therapeutic implications. *Br. Med. Bull.*, **39**, 271.

CAMPBELL, F., HAYNES, J., EVANS, D. & 4 others (1983). Prostaglandin $E_2$ synthesis by tumour epithelial cells and oestrogen receptors (ER) status of primary breast cancer. *Clin. Oncol.*, **9**, 75.

ELIAS, E., SEPULVEDA, F. & MINK, I. (1973). Increasing the efficiency of cancer chemotherapy with heparin: Clinical Study. *J. Surg. Oncol.*, **5**, 189.

GASIC, G., GASIC, T., GALANTI, N., JOHNSON, T. & MURPHY, S. (1973). Platelet-tumour cell interactions on mice. The role of plateles in the spread of malignant disease. *Int. J. Cancer*, **11**, 704.

GRANSTROM, E. & KINDAHL, H. (1978). Radioimmunoassay of prostaglandins and thromboxanes. *Adv. Prostagl. Thromboxane Res.*, **5**, 119.

GREAVES, M., IBBOTSON, K., ATKINS, D. & MARTIN, T. (1980). Prostaglandins as mediators of bone resorption in renal and breast tumors. *Clin. Sci.*, **58**, 201.

HAMBERG, M., SVENSSON, J. & SAMUELSSON, B. (1975). Thromboxanes: A new group of biologically active compounds derived from prostaglandin endoperoxides. *Proc. Natl. Acad. Sci. USA*, **72**, 2994.

HONN, K.V., CICONE, B. & SKOFF, A. (1981). Prostacyclin: A potent metastatic agent. *Science*, **212**, 1270.

HUMES, J., BONNEY, R., PELUS, L. & 4 others (1977). Macrophages synthesize and release prostaglandins in response to inflammatory stimuli. *Nature*, **269**, 149.

KARMALI, R., WELT, S., THALER, H. & LEFEVRE, F. (1983). Prostaglandins in breast cancer: Relationship to disease stage and hormone status. *Br. J. Cancer*, **48**, 689.

KIBBEY, W., BRONN, J. & MINTON, J. (1979). Prostaglandin synthetase and prostaglandin $E_2$ levels in human breast carcinoma. *Prostaglandins Med.*, **2**, 133.

LIONE, A. & BOSMAN, H. (1978). The inhibitory effect of heparin and warfarin treatments on the intravascular survival of B16 melanoma cells in syngeneic C57 mice. *Cell Biol. Int. Rep.*, **2**, 81.

MALACHI, T., CHAIMOFF, C., FELLER, N. & HALBRECHT, I. (1981). Prostaglandin $E_2$ and cyclic AMP in tumor and plasma of breast cancer patients. *J. Cancer Res. Clin. Oncol.*, **102**, 71.

MONCADA, S., GRYGLEWSKI, R., BUNTING, S. & VANE, J.R. (1976). An enzyme isolated from arteries transforms endoperoxides to an unstable substance that inhibits platelet aggregation. *Nature*, **263**, 663.

PESKAR, B.M., WEILER, H., SCHMIDBERGER, P. & PESKAR, B.A. (1980). On the occurrence of prostacyclin metabolites in plasma and vascular tissue as determined radioimmunologically. *Febs Letters*, **121**, 25.

ROLLAND, P., MARTIN, P., JACQUEMIER, J., ROLLAND, A. & TOGA, M. (1980). Prostaglandin in human breast cancer. Evidence suggesting that an elevated prostaglandin production is a marker of high metastatic potential for neoplastic cells. *J. Natl Cancer Inst.*, **64**, 1061.

SLOANE, B., DUNN, J. & HONN, K. (1981). Lysosomal cathepsin B: Correlation with metastatic potential. *Science*, **212**, 1151.

SOKAL, R.R. & ROHLF, F.J. (1981). Assumptions of analysis of variance. In: *Biometry* (Eds. Sokal & Rohlf), 2nd Ed., W.H. Freeman and Co., San Francisco, p. 450.

VERGOTE, I.B., LAEKEMAN, G.M., KEERSMAEKERS, G.H. & 6 others (1985). Prostaglandin $F_{2\alpha}$ in benign and malignant breast tumours. *Br. J. Cancer*, **51**, 827.


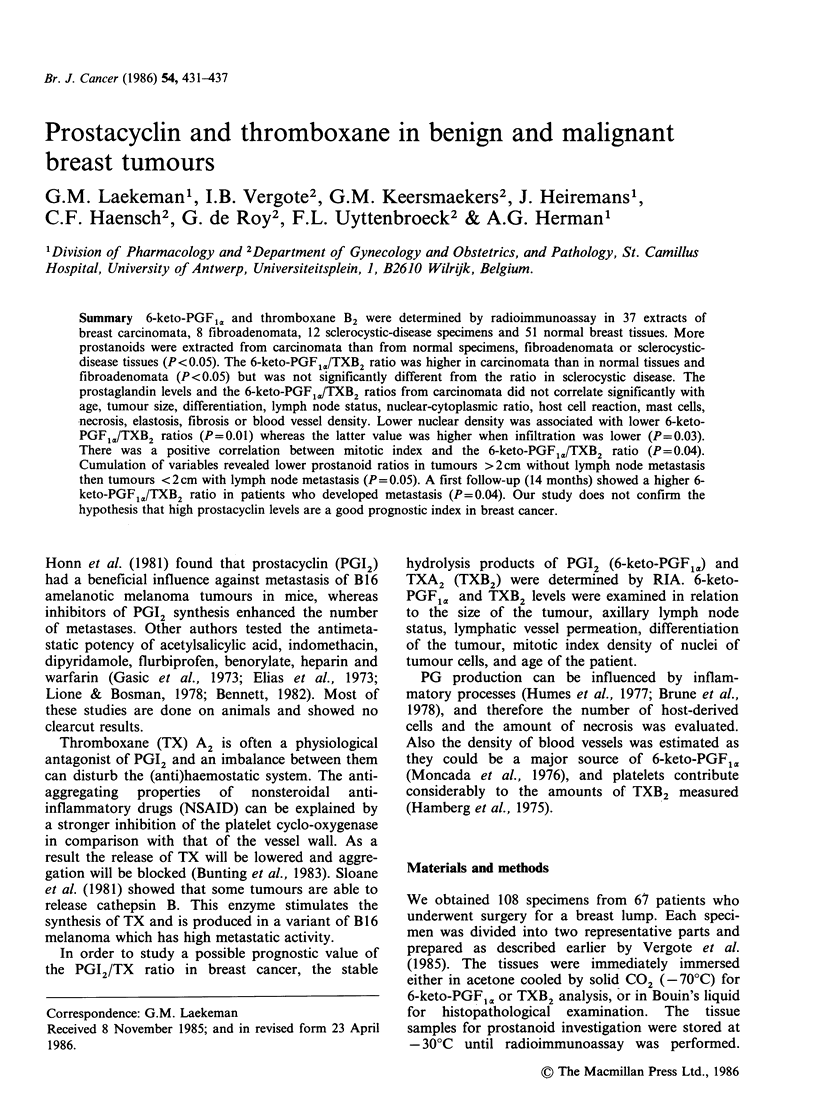

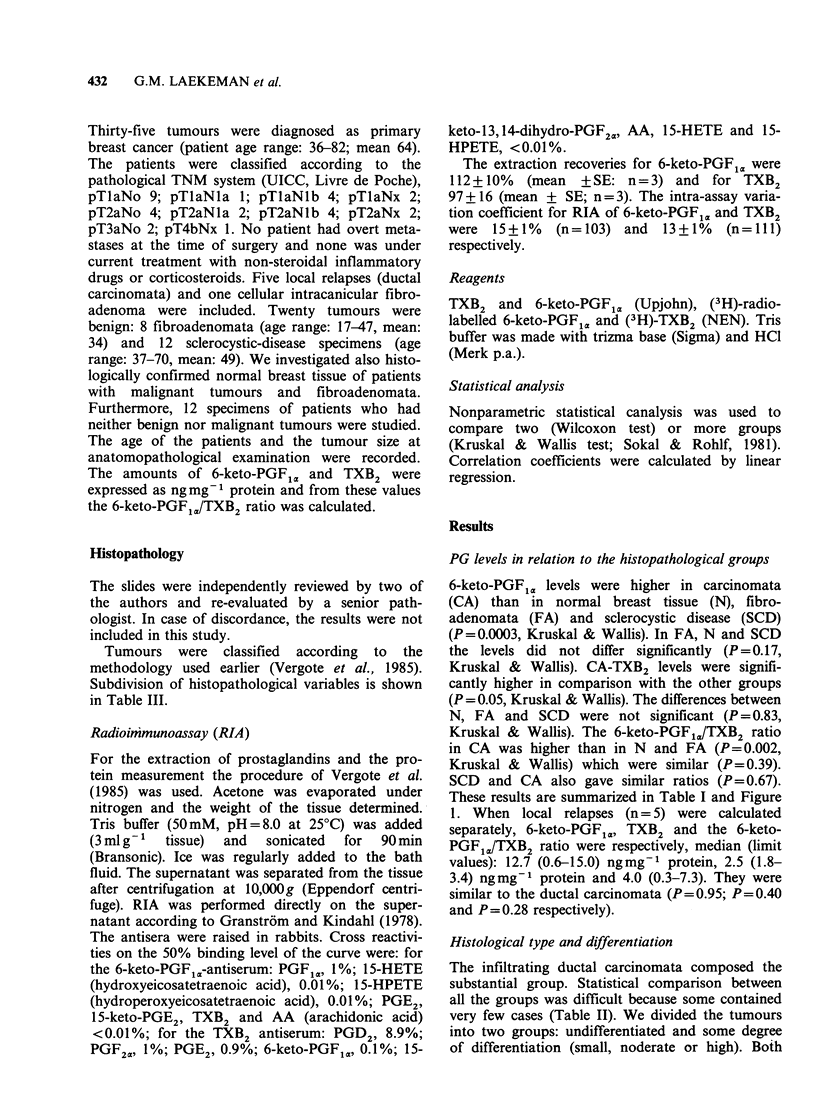

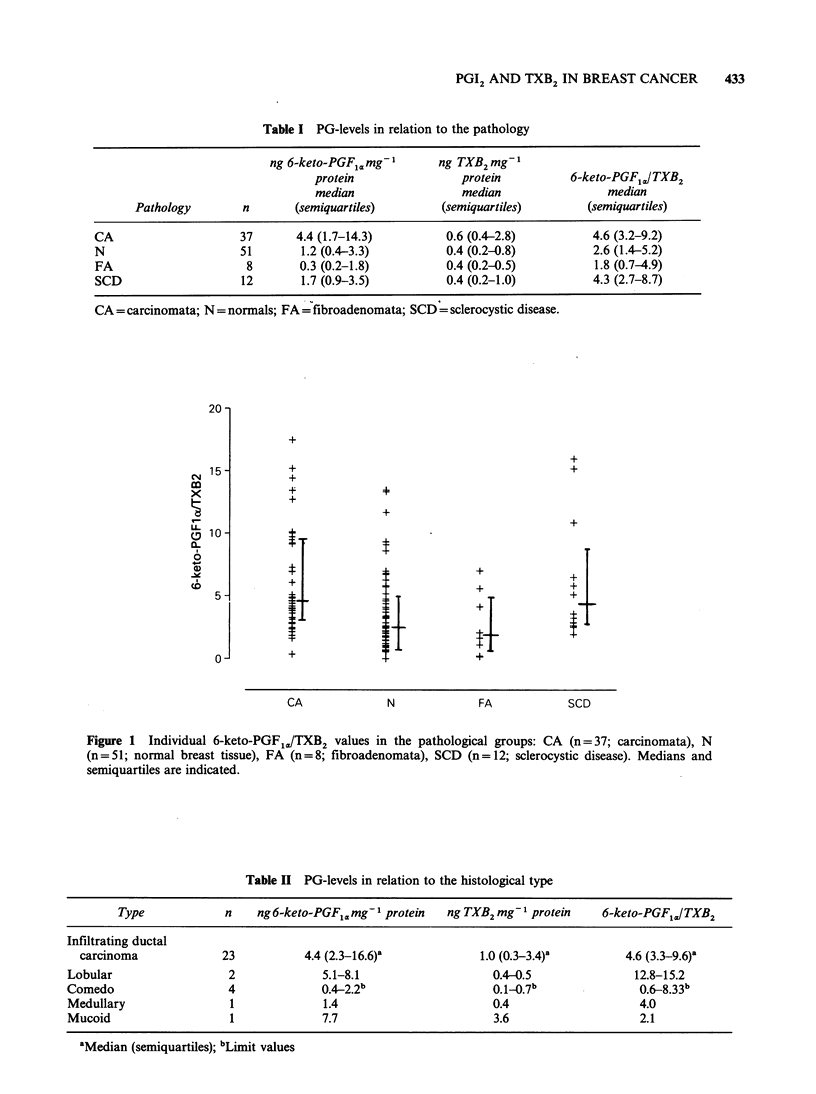

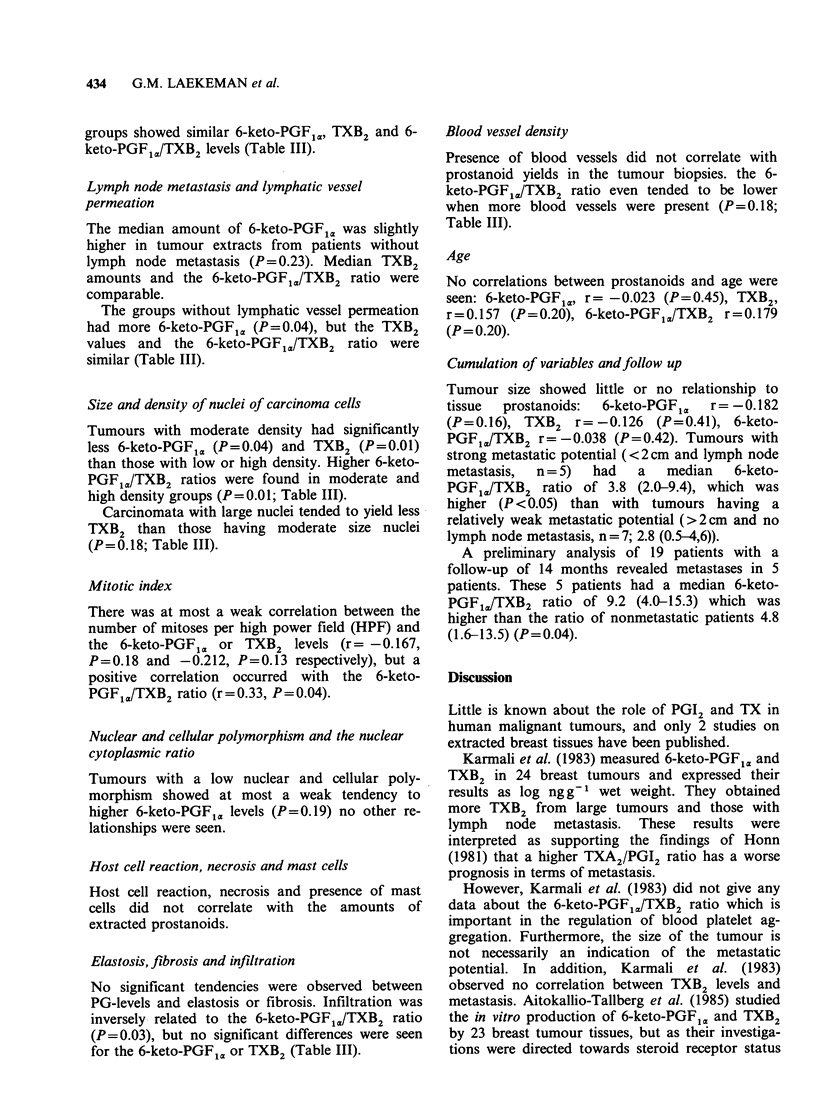

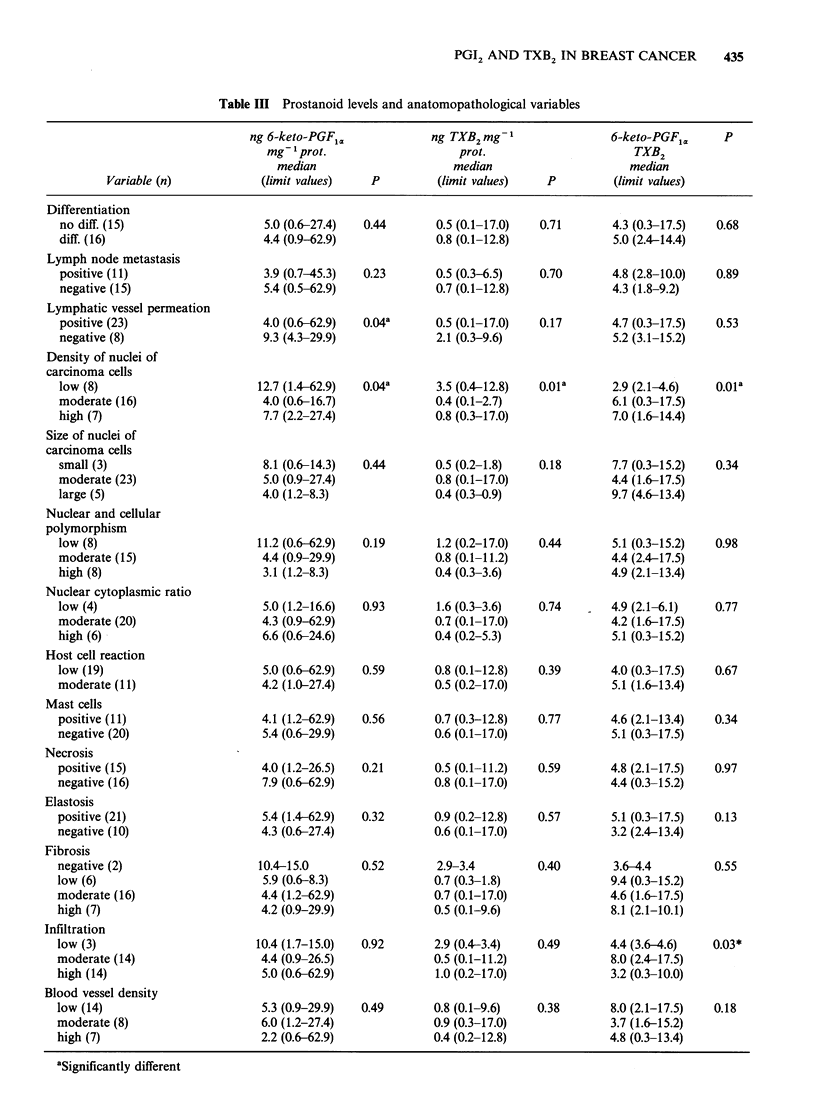

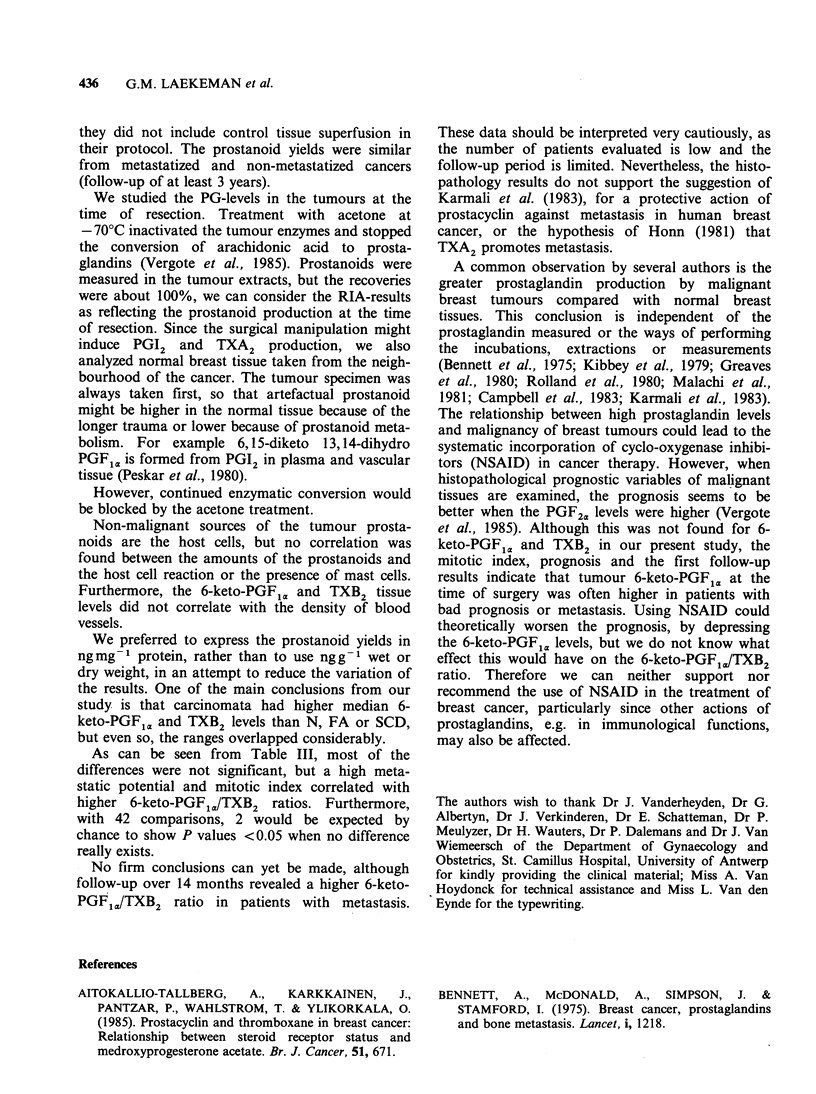

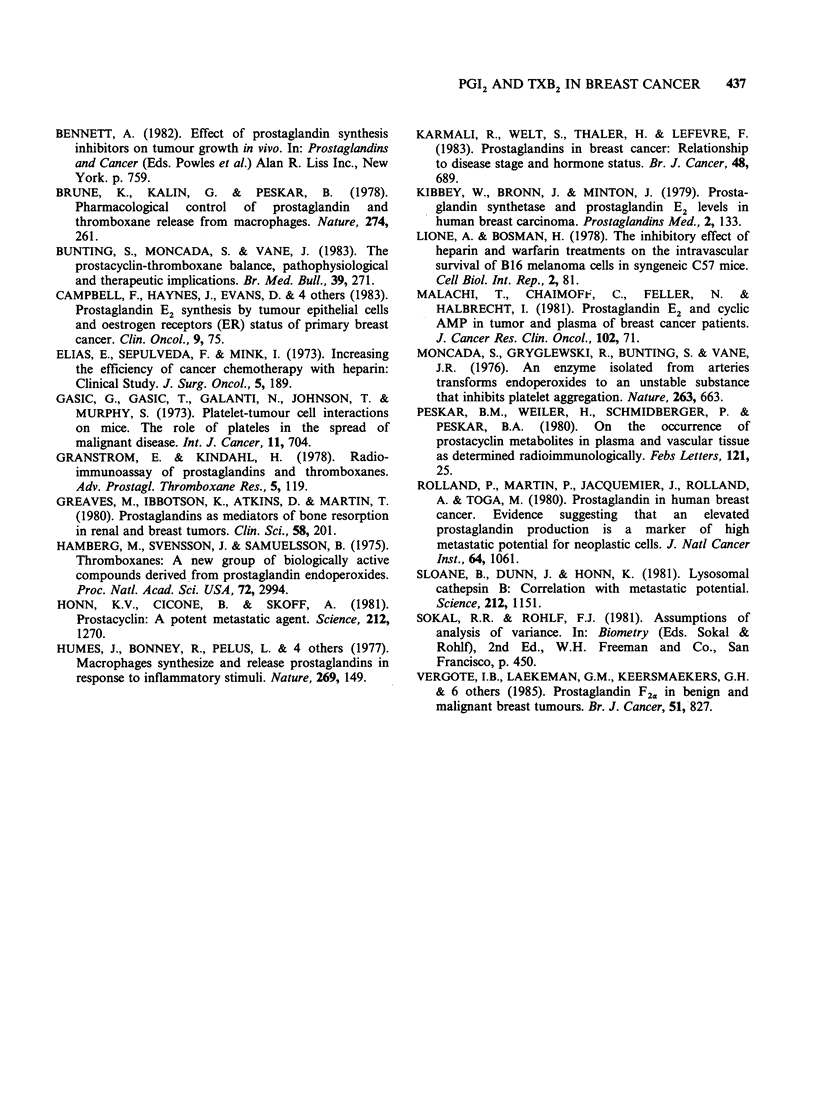


## References

[OCR_00842] Aitokallio-Tallberg A., Kärkkäinen J., Pantzar P., Wahlström T., Ylikorkala O. (1985). Prostacyclin and thromboxane in breast cancer: relationship between steroid receptor status and medroxyprogesterone acetate.. Br J Cancer.

[OCR_00849] Bennett A., McDonald A. M., Simpson J. S., Stamford I. F. (1975). Breast cancer, prostaglandins, and bone metastases.. Lancet.

[OCR_00862] Brune K., Glatt M., Kälin H., Peskar B. A. (1978). Pharmacological control of prostaglandin and thromboxane release from macrophages.. Nature.

[OCR_00868] Bunting S., Moncada S., Vane J. R. (1983). The prostacyclin--thromboxane A2 balance: pathophysiological and therapeutic implications.. Br Med Bull.

[OCR_00879] Elias E. G., Sepulveda F., Mink I. B. (1973). Increasing the efficiency of cancer chemotherapy with heparin: "clinical study".. J Surg Oncol.

[OCR_00884] Gasic G. J., Gasic T. B., Galanti N., Johnson T., Murphy S. (1973). Platelet-tumor-cell interactions in mice. The role of platelets in the spread of malignant disease.. Int J Cancer.

[OCR_00890] Granström E., Kindahl H. (1978). Radioimmunoassay of prostaglandins and thromboxanes.. Adv Prostaglandin Thromboxane Res.

[OCR_00895] Greaves M., Ibbotson K. J., Atkins D., Martin T. J. (1980). Prostaglandins as mediators of bone resorption in renal and breast tumours.. Clin Sci (Lond).

[OCR_00900] Hamberg M., Svensson J., Samuelsson B. (1975). Thromboxanes: a new group of biologically active compounds derived from prostaglandin endoperoxides.. Proc Natl Acad Sci U S A.

[OCR_00906] Honn K. V., Cicone B., Skoff A. (1981). Prostacyclin: a potent antimetastatic agent.. Science.

[OCR_00911] Humes J. L., Bonney R. J., Pelus L., Dahlgren M. E., Sadowski S. J., Kuehl F. A., Davies P. (1977). Macrophages synthesis and release prostaglandins in response to inflammatory stimuli.. Nature.

[OCR_00916] Karmali R. A., Welt S., Thaler H. T., Lefevre F. (1983). Prostaglandins in breast cancer: relationship to disease stage and hormone status.. Br J Cancer.

[OCR_00922] Kibbey W. E., Bronn D. G., Minton J. P. (1979). Prostaglandin synthetase and prostaglandin E2 levels in human breast carcinoma.. Prostaglandins Med.

[OCR_00927] Lione A., Bosmann H. B. (1978). The inhibitory effect of heparin and warfarin treatments on the intravascular survival of B16 melanoma cells in syngeneic C57 mice.. Cell Biol Int Rep.

[OCR_00933] Malachi T., Chaimoff C., Feller N., Halbrecht I. (1981). Prostaglandin E2 and cyclic AMP in tumor and plasma of breast cancer patients.. J Cancer Res Clin Oncol.

[OCR_00939] Moncada S., Gryglewski R., Bunting S., Vane J. R. (1976). An enzyme isolated from arteries transforms prostaglandin endoperoxides to an unstable substance that inhibits platelet aggregation.. Nature.

[OCR_00945] Peskar B. M., Weiler H., Schmidberger P., Peskar B. A. (1980). On the occurrence of prostacyclin metabolites in plasma and vascular tissue as determined radioimmunologically.. FEBS Lett.

[OCR_00952] Rolland P. H., Martin P. M., Jacquemier J., Rolland A. M., Toga M. (1980). Prostaglandin in human breast cancer: Evidence suggesting that an elevated prostaglandin production is a marker of high metastatic potential for neoplastic cells.. J Natl Cancer Inst.

[OCR_00960] Sloane B. F., Dunn J. R., Honn K. V. (1981). Lysosomal cathepsin B: correlation with metastatic potential.. Science.

[OCR_00971] Vergote I. B., Laekeman G. M., Keersmaekers G. H., Uyttenbroeck F. L., Vanderheyden J. S., Albertyn G. P., Haensch C. F., De Roy G. J., Herman A. G. (1985). Prostaglandin F2 alpha in benign and malignant breast tumours.. Br J Cancer.

